# What Social Supports Are Available to Self-Employed People When Ill or Injured? A Comparative Policy Analysis of Canada and Australia

**DOI:** 10.3390/ijerph19095310

**Published:** 2022-04-27

**Authors:** Tauhid Hossain Khan, Ellen MacEachen, Debra Dunstan

**Affiliations:** 1 School of Public Health Sciences, University of Waterloo, Waterloo, ON N2L 3G1, Canada; ellen.maceachen@uwaterloo.ca; 2 Department of Sociology, Jagannath University, Dhaka 1100, Bangladesh; 3 School of Psychology, University of New England, Armidale, NSW 2350, Australia; ddunstan@une.edu.au

**Keywords:** precarious work, self-employed, work injury, work disability, social security, social support, Australia, Canada

## Abstract

Self-employment (SE) is a growing precarious work arrangement internationally. In the current digital age, SE appears in configurations and contours that differ from the labor market of 50 years ago and is part of a ‘paradigm shift’ from manufacturing/managerial capitalism to entrepreneurial capitalism. Our purpose in this paper is to reflect on how a growing working population of self-employed people accesses social support systems when they are not working due to injury and sickness in the two comparable countries of Canada and Australia. We adopted ‘interpretive policy analysis’ as a methodological framework and searched a wide range of documents related to work disability policy and practice, including official data, legal and policy texts from both countries, and five prominent academic databases. Three major themes emerged from the policy review and analysis: (i) defining self-employment: contested views; (ii) the relationship between misclassification of SE and social security systems; (iii) existing social security systems for workers and self-employed workers: Ontario and NSW. Our comparative discussion leads us toward conclusions about what might need to be done to better protect self-employed workers in terms of reforming the existing social security systems for the countries. Because of similarities and differences in support available for SE’d workers in the two countries, our study provides insights into what might be required to move the different countries toward sustainable labour markets for their respective self-employed populations.

## 1. Introduction

Self-employment (SE) has emerged as a non-standard, precarious, and contingent work relationship internationally [1,2,3]. The proportion of precarious work, including SE, has been growing rapidly in recent decades due to globalization, dramatic technological advances, the information revolution, and the decline of manufacturing industries [2,4,5]. It has been estimated that non-standard employment accounts for more than 60% of workers worldwide [6,7]. This trend is accelerated by the rising ‘gig’ economy, which is undermining traditional employment relations with secure jobs, predictable, advancement, and stable pay [8,9,10,11]. Of importance, Self-employed (SE’d) workers now comprise 15% of employment in Europe [12] and 10% of the Australian workforce [13]. In Canada, 2.9 million people were SE’d in 2018, double the number in 1976 [14], although this increasing trend has remained stable in Canada for the last decade. In general, women, recent immigrants, and other visible minorities tend to choose SE to meet their needs that derives from traditional social roles (e.g., women as a caregiver and to earn money to support their families, immigrants due to lack of suitable paid jobs) [2].

Mounting international evidence stresses that precarious employment conditions are having profound adverse effects on workers’ safety, health, and wellbeing [15,16,17]. Despite this, SE’d workers are one of the ambiguous categories of working groups who are largely excluded from the workers’ compensation coverage internationally [4]. However, research on SE’d people in terms of their access to social supports systems when they are not working due to injury and sickness is scarce, and, as a consequence, policies geared towards building inclusive workers’ compensation policies, upgrading the social safety net programs, and reforming statuary/legal frameworks often ignore complex interactions and responses within them.

Our purpose in this paper is to reflect on how a growing working population of SE’d people access welfare state social support systems when they are away from work due to injury and sickness in the two comparable countries of Canada and Australia- in terms of social, political, and cultural contexts. We take the cases of Canada and Australia, as both countries have key similarities in terms of comparable economies and liberal welfare states [18]. As well, both have social welfare policies that differ by state/province, and each addresses occupational illness and injury via workers’ compensation systems. They also have a similar penetration of the new ‘gig’ economy and a similar proportion of SE’d workers, accounting for around 8–10% of employed people in 2016 [19]. Because of these similarities, a comparative analysis is useful for understanding actions that can support greater sustainability of labour markets and economies of their respective SE’d populations.

In this paper, we make three distinct contributions. First, we explore how challenges to defining the status of a worker/SE are connected to accessibility to social supports in general and comparative analysis of two jurisdictions of Australia and Canada that recognize differences (similarities as well) in their social support policies and legal protocols. Second, we unpack the debates around the definitions, classifications, and misclassifications of SE, shedding light on differences between the two countries, convergence and divergence of different stakeholders’ views and perspectives, showing how they define, redefine, and reform the status of SE for the sake of their socio-political interest. Third, we make a snapshot of the social support systems available for workers for the said jurisdictions, where the status and position of SE’d workers are conspicuously designated, by analysing when SE’d workers are entitled to the available support systems, can opt out or opt into the supports; this analysis demonstrates the relative strengths and limitations, and gaps of the existing systems, which provides lessons for both jurisdictions for further policy formulation and reformations. Finally, the paper concludes with policy implications, as this study is prescriptive in nature, that is, it follows a method of analysis aimed at new policy ideas in order to improve the social welfare of SE’d workers in Canada and Australia [20]. In the context, mentioned above, our analysis was guided by ‘interpretive policy analysis’, focusing on meaning-making processes that are contextual, and situation-specific, instead of focusing on general laws or universal principles [21,22].

## 2. Literature Review

### 2.1. Dynamics of SE’d Workers

SE’d workers are generally depicted as a special group of homogenous people [5], who possess good health, enjoy the freedom of being their own boss and flexible working hours, do not rely on social security protection, and enjoy greater job satisfaction and improved quality of life [12,23]. They are also described as taking on a high level of personal risk to grow their businesses and creating employment opportunities for others [5,9,12,17]. However, these depictions do not reflect the recent reality of the SE’d [12]. Surprisingly, very few attempts have been made in order to investigate systematically how these new forms of employment impact the SE’d in the face of occupational injury and disease [4]. This is despite a growing body of research that argues that the rise of precarious employment, including outsourcing, downsizing, and small business, adversely affects workers’ occupational health and safety [24,25,26,27,28].

A clear dark side of this SE labour market exists in that a significant number of SE’d workers are compelled to undertake this type of work due to unemployment, scarcity of alternatives, and everyday financial hardships [2,5,12]. As argued by The Law Commission of Ontario [2], all SE’d workers should not be treated in the same manner: “The experiences and vulnerabilities of this group range from billionaire entrepreneurs to taxi drivers working 90 h a week simply to pay their bills and includes many people who are gaining income from self-employment activity alongside their main job” (p.75). As such, SE does not always mean self-sufficiency. Instead, some SE’d workers, with low earnings, are precarious workers at risk of poverty and social exclusion [29].

In addition to income-based poverty, a key challenge facing SE’d workers is what happens when they are unable to work due to illness or injury/disabilities, whether on a short- or long-term basis. This is also connected to poverty but in a different fashion. Some SE’d workers do not expect sick pay, paid annual leave, or a future pension because they are well-off and have adequate savings for the future [17]. Some literature stresses that low-income SE can have a considerable impact on workers’ physical, social, and personal lives (e.g., family relations), promoting a greater risk of injury, illness, stress, and challenges to health care access [2,11,30,31]. Mounting evidence also shows a strong relationship between the precarious job and poorer health outcomes [32], and greater social costs such as the undermining of intimate relationships [15,33]. As well, SE’d workers are at higher risk for certain diseases compared to salaried workers [17]. However, SE’d workers are less likely to purchase health insurance policies in the USA, which may affect their health and wellbeing if they use little or inappropriate medical care [17,34].

### 2.2. Social Security Systems Protecting SE’d Workers: The Inclusion/Exclusion Game

Globally, many policies and much legislation, such as workers’ compensation, employment insurance, and state pension plans, exclude SE’d workers. Indeed, Quinlan [4] noted that SE’d workers are fully excluded from most countries’ workers’ compensation coverage policies. In some countries (e.g., Estonia, Latvia, Portugal, and the Slovick Republic), 40–50% of precarious workers are less likely to receive any form of income support when they are out of work due to injury, sickness, or any form of impairment [10]. The ILO’s (2020) study of G20 countries found a social protection coverage gap for SE’d workers in many of the countries [19]. This report recommended several measures to protect the SE’d, including preventing the false classification of workers as SE’d and reducing the ‘grey zone’ of vague employment status [19]. However, some welfare states play pivotal roles in terms of protecting SE’d workers. For example, Finland provides a broad support system to workers regardless of employment status, in which SE’d workers are covered with earnings-related pension schemes (old-age pension, disability pension, survivors’ pension) and have access to a universal basic social security system (parental and sickness benefits, housing, and unemployment benefits) [35]. In the UK, there was a ‘policy vacuum’ observed in social security policy for SE’d people in the 1980s; however, SE’d people were included in state insurance systems and mainstream income-related benefits as of the 1990s [36]. Despite this, they are still excluded from many benefits systems in the UK, such as income supports, housing benefits, council tax benefits, family credit, and disability working allowances, due to administrative weakness [36]. The British perspectives are consistent with Finland’s estimation that there is a gulf between tax declared-income and pension declared-income scheme for self-employed workers (under-insurance) within the statutory pension; they pay too little to contributions, leading to inadequate protection against personal risks [35].

Spasova, et al. [37] illustrated an interesting correlation between SE’d people’s access to statuary social protection systems and types of welfare regimes in 35 European countries. They reveal that in countries with social democratic regimes (e.g., Finland, Denmark, Iceland, Norway, Sweden) where social protections depend on ‘general taxation’, the SE’d workers have access to all statuary schemes and are treated as salaried workers. They are also treated in a similar manner in the Liberal regime countries (e.g., Ireland and UK) in terms of social protection for the self-employed worker. However, the countries whose schemes rely on ‘heavy taxations’ make distinctions between salaried and self-employed workers in terms of access to social protections; while salaried workers can access both means-tested benefits and insurance-based benefits, SE’d workers can access means-tested, but often at a low level. Interestingly, some countries, such as the Corporatist (Austria, Belgium, and Germany) and Southern European regimes (Italy, Spain), show a variance in statutory access to social protections, including insurance schemes, and these differences not only exist between SE’d and salaried, but also within different SE’d patterns. In our view, this study shed new light on (which previous studies had not addressed), the uneven access to statutory social protections being brought about by the complicated and robust dynamics of SE’d themselves in terms of their actions and nomenclature.

Overall, Spasova et al.’s analysis shows that although the welfare countries show comparatively comprehensive social protection for self-employed people in terms of the access to (basic) pension and (basic) health insurance, they still have social protection coverage gaps for SE’d in countries [38]. To put it another way, although welfare economies are supportive of protecting SE’d workers, they still struggle with administrative and bureaucratic shortcomings in terms of supporting SE’d workers with social protections. As such, this exclusion of SE’d workers advances a central question to the agencies, employers, policymakers, government stakeholders, and workers: how do the established norms and existing legislative protocols fit with the changing labour market [39], with the special reference to SE? However, without social safety nets, many lower-income SE’d workers are unable to ensure their house rent, medical costs, food, and future security (e.g., retirement pension). Similar to employees in standard employment, they may encounter the same level of anxiety, stress, and illness due to being in work or when out of work. In this context, the absence of a social safety net can perpetuate their distress.

Although a growing body of research sheds light on SE’d workers in terms of their health and well-being, social mobility, and racial and gender discrimination [1,31,40,41,42,43], very few research or policy reports consider SE’d workers in terms of their social security and supports [2,5,44]. Moreover, with some exceptions [45], a focus on work disability of SE’d workers in legislation (e.g., labour laws), policy (e.g., workers ‘compensation), and academic research has been largely ignored. As such we know little about the role of government and policymakers in terms of providing supports to SE’d workers [45].

## 3. Methods

We adopted ‘interpretive policy analysis’ [21] as a methodological framework, which is a widely used approach for policy analysis or policy research [22,46,47] and involves analyzing public policies, as a form of text or representation of social actions. This approach focuses on contexts and meaning-making processes that are situation-specific, instead of focusing on general laws or universal principles [22]. This approach then helps us to interpret and establish relationships between different issues, develop arguments, and eventually draw a cogent conclusion.

We collected and analysed a range of secondary data related to work disability policy and practice in Canada and Australia. We focused on ‘work disability policy’, which is diverse policies connected to workers’ compensation, sickness and disability policy, and the legal and regulatory protocols and frameworks of social security [39]. The search for documents was performed in several phases. Official data, legal, and policy texts from both countries were used (i.e., material generated by governments and their agencies). These were identified using the Google search engine and by visiting libraries of the two universities—the University of Waterloo and the University of New England in Canada and Australia (Table 1). Apart from the established databases, Google was used because it is a popular tool for seeking specific information and relevant outcomes for a typical query [48]. In addition, observations and commentaries (e.g., updated statistics) from global agencies such as the World Health Organization, the World Bank, and the International Labour Organisation were utilized (Table 2). Then, the lead author identified possible peer-reviewed literature through a systematic search of five databases including PubMed, SCOPUS, PSYCHINFO, ABI/INFORM, AND CINAHL (See Appendix A for Keywords). A review of titles and abstracts for articles relevant to SE, work injury, and return to work was conducted. In all, 22 articles were identified as relevant (Table 2). Of these, three articles (one for Canada, two for Australia) focused on Australia and Canada. After that, the lead author searched (the second search) the SCOPUS database separately, focusing on Australia and Canada (See Appendix A for keywords). Of 93 documents identified, three articles were relevant to our study. Finally, we also searched ‘Google scholar’ and ‘google.com’ separately using refined and specific key terms related to Canada, Ontario, Australia, and NSW, including SE in Canada/Australia, SE in Ontario/NSW, workers compensation in Canada/Australia, employment Insurance in Canada/Ontario, personal accident insurance in NSW, in order to get more specific peer-reviewed articles and grey literature related to Canada and Australia. This resulted in seven relevant documents (out of 144) for inclusion in our synthesis (Table 1).

The final selected documents obtained from both searches-systematic and non-systematic were examined following Dixon-Woods and colleagues’ processes of quality assessment, data extraction, and data synthesis [58,59]. They underline the importance of assessing the quality of the articles to be included in the review and analysis in terms of their overall relevance to facilitating understanding of the topic under study [3]. Systematic data extraction focused on demographic information, research questions, the purpose of the study/report/review, year of publication, place of publication, methods, main findings, and sector of SE. This approach resulted in a comprehensive overview of the selected articles and documents and facilitated analytical exchanges between the authors. A summary description of the documents is in Table 1 and Table 2. Data were synthesized by recurring concepts, which ultimately contributed to themes. A process of constant comparison and negative case analysis guided the synthesis, which involved assembling issues and grouping topics under common areas. For example, authors might use dissimilar words, but be addressing a similar general concept (e.g., SE, independent contractor). The negative case analysis focused on studies that appeared to contradict each other. For instance, the Canada Employment Insurance Commission (2014) reported that SE’d women (25 and 44 years) made 90.4% of all special benefits claims, mostly for maternity and parental benefits. However, according to Hilbrecht [30], a significant number of entitled SE’d workers, irrespective of gender, do not seek and claim compensation mainly due to a lack of information about the supports [30]. In these cases, we attempted to reconcile these contradictions by noting contexts and methods. In this example, the negative case analysis directed attention to the reasons why poor benefit claimant rates among SE’d exist, which provided insight into weaknesses in existing policies with supporting SE’d workers in both Canada and Australia. This research followed three phases of synthesis leading to the final themes. First, an open-coding system was used to analyze the documents. This helped us to reflect on the overall patterns of our data, including identifying the repeated and common themes. In the second phase, open codes were re-reviewed and focused codes were generated. A focused code is a pattern or category that groups two or more open codes [77]. Our focused codes then led to three major themes, together with sub-themes, focused on: (i) defining self-employment: contested views; (ii) the relationship between misclassification of SE and social security systems; (iii) existing social security systems for workers and SE’d workers: Ontario and NSW. The lead author met and consulted with senior authors on a regular basis to discuss ongoing analyses of findings and to challenge preliminary interpretations, which facilitated thorough interpretations of the findings. 

**Table 2:** Description of literature identified by the systematic search.

Articles, Year (Reference)	Country	Method	Major Findings
McNaughton, et al. [78]	USA	Quantitative	-Vocational rehabilitation counselors and support personnel should advocate for an appropriately challenging educational program -Vocational rehabilitation and support personnel can offer an important work-place perspective on the individual’s communication skills and priorities for intervention -Vocational rehabilitation counsellors and support personnel should help identify a wide variety of part-time or ‘work-experience’ jobs while the individual who uses AAC is still in school.
Arnold and Ipsen [79]	USA	Policy analysis	-Unlike in the past, when counsellors assumed a great deal of responsibility for developing the business or writing the plan, now the counsellor usually facilitates the process, and the consumer develops the business and business plan with the help of external business developers. Most state agencies will not support development of a nonprofit business.
Larson and Hill [80]	USA	Quantitative	-SE’d adults and those working in small establishments are less likely to be offered insurance. -Only in the most rural area does working in agriculture, fishing, and forestry have a statistically significant effect, controlling for other factors such as self-employment.
Hartman, et al. [81]	Netherlands	Quantitative	-In the Netherlands, there is no social insurance for SE’d persons during the first year of sick leave. After 1 year of sick leave, social insurance provides compensation for loss of income to a maximum of 70% of the statutory minimum income. -This financial gap can be bridged by an insurance policy. -An estimated 63% of self-employed farmers take out an insurance policy with a private insurance company, which provides supplementary compensation for loss of income if they are unable to work due to illness or an accident.
Rizzo [82]	USA	Policy analysis	-Identifying the supports an individual may need in the employment setting requires a critical and unabashed look at skills and capacities. Essential to this process is the inclusion of the consumer in all aspects of need-assessment, decision-making, and plan development. -Opportunities to manage the business and perform business-related tasks allows the consumer to develop SE skills, as long as these are truly managerial and decision-making in nature.
Fossen and König [83]	Germany	Quantitative	-Those who enter into SE are more often male, have had a SE’d father, and are more willing to take risks than the other paid employees. -They are more often active in the business services and construction industries and less often in manufacturing and public and personal services. -The health insurance system may provide incentives to enter SE for persons whose income is not high enough to opt out of the SHI as a paid employee. For them, self-employment lifts the barrier to PHI.
Hilbrecht [30]	Canada	Qualitative	-Many were unaware of EI special benefit program, which provided maternity leave, parental leave, compassionate care leave, sickness benefits, and benefits for parents of critically ill children to self-employed people. -Different types of informal support often existed simultaneously: family support, spousal support (emotional and income support). -Some women expressed gendered assumptions about men as providers who could offer a financial safety net if their business floundered.
Barber III and Moffett [84]	USA	Quantitative	-The probability that a SE’d individual in a state that had implemented a subsidy would be covered by private insurance increased by about 4 percentage points after the subsidies were implemented when compared to the self-employed in the control states. -The subsidies were not enough to increase the probability that an individual in the treatment states after the policies would decide to become SE’d. -The determinants of the choice to become SE’d involve much more than the cost of health insurance.
Grégoris, et al. [85]	France	Quantitative	-SE’d workers have a higher morbidity than employees. Conversely, the SE’d group had greater task variation, which might reduce morbidity effects. -The lack of occupational health services also contributes to this difference. -Need for occupational health services for self-employed workers, with occupational health surveillance and prevention strategies in order to reduce occupational risks.
Sharp, Torp, Van Hoof and de Boer [12]	European region	Commentary	Evidence is lacking on how best to support SE’d survivors to (re-)engage with work or business after cancer. Most interventions to enhance cancer survivors’ work outcomes have been pertinent (only) for salaried employees and have focused on return to work.
Wijnvoord, et al. [86]	Netherlands	Quantitative	-Higher educated SE’d showed that the hazard of experiencing a new period of sickness absence increased with every previous period. This effect was found for both sexes and also for most diagnostic categories of the first period of sickness absence. -Musculoskeletal disorders and mental and behavioural disorders were the most frequent causes of long-term sickness absence. -Locomotor disorders were more frequent, but mental disorders lead to longer duration of sickness absence.
Ashley and Graf [87]	USA	Quantitative	-Causes for choosing SE: a lack of decent wages and promotion opportunities, for intolerance of mental illness symptoms such as panic attacks, anxiety, and depression; difficulty in obtaining work accommodations; long hours; and being let go due to disability. -Participants noted their health challenges were easier to manage when self-employed, and they experience lower levels of stress and greater flexibility.
Ostrow, et al. [88]	USA	Quantitative	-SE is acting as a financial bridge or means of exploring career opportunities. -Most respondents had not accessed Social Security’s back to work programs. -While SE’d individuals struggle to access these benefits, they also have better access, or find these programs more attractive, than individuals with psychiatric disabilities seeking wage employment.
Quinlan [15]	Australia	Qualitative	-17.7% of the workforce mainly are SE’d (two-thirds of whom are concentrated in four industries: agriculture, fishing and forestry; construction; retail; and property and business services), unpaid helpers and volunteers–were not covered by workers’ compensation. -Where workers were deemed to be SE’d subcontractors by industrial relations and taxation law, they presumed they were denied workers’ compensation. -Another problem determining eligibility occurred where workers changed employment status (e.g., from employee to self-employed or small employer and then back) on a regular basis (in response to aspirations or bankruptcy, principal contractor demands or shifts in the business cycle).
Rietveld, Van Kippersluis and Thurik [17]	USA	Quantitative	-SE is, to a certain extent, influenced by genetic factors. It is perceivable that the same genetic factors influence both SE and health (such a mechanism is called pleiotropy genetics)
Gevaert, De Moortel, Wilkens and Vanroelen [31]	European regions	Quantitative	-Farmers and dependent freelancers and own account workers have worse mental well-being than medium to big employers. -Entrepreneurial characteristics are able to explain mental well-being differences between types of SE’d -Country-level perception of entrepreneurs influences their mental well-being.
Beattie, et al. [89]	Australia	Qualitative	SE’d farmers are often not covered by workers’ compensation insurance and therefore, if they have not purchased their own income protection policy, have no means for receiving financial assistance during the recovery phase.
Yoon and Bernell [16]	USA	Quantitative	SE’d individuals in the US are physically healthy, or healthier than wage-earners, despite the relative lack of health insurance among SE’d persons as compared to wage-earning persons. -No significant relationship between SE and mental health. -Individuals do not experience a greater barrier of access to necessary health care, despite a higher rate of being uninsured among SE’d individuals in the US, the SE’d may be able to finance their own health care using their incomes or accumulated savings. -SE’d are more likely than wage-earning individuals to engage in health- promoting activities, perhaps due to greater flexibility in making room for health promotion activities into their schedule.

## 4. Findings

### 4.1. Defining Self-Employment: Contested Views

Prevailing definitions and conceptualizations of SE are contested and vary, which reflects that there is not one type or state of SE. Additionally, the existing legal protocols, in Canada and Australia, dealing with employee and employment minimally defines SE, as is shown in the Table 3. There is a debate around SE and whether it brings benefits or barriers for sustainability in terms of health [17], facilitates life-work balance [30] and is adequate in terms of income [90]. Different stakeholders pertinent to employment, tax and revenue management, workers’ compensation management, social supports agencies, judiciaries, politicians, public policy makers, researchers, and academics have been defining SE and naming this employment system from a variety of perspectives. The intentions and motivations differ behind these differing views, as they are derived from political (e.g., political public policy), ethical (e.g., social justice), and philosophical (e.g., neoliberal agenda) grounds. Thus, available literature [4,49,54,62,91,92] uses different names for SE interchangeably as depicted in Table 4.

These multiple terms make SE challenging to define, both conceptually and empirically [30]. According to Cohen, Hardy and Valdez (2019), SE is not a fixed category/pattern and is contingent on changing structural relationships, which are subject to the mode of production and economy (e.g., manufacturing, service, and digital economy, or labour market, and economic status of society) [92]. For example, during the (2007/2008) global economic recession, three patterns of SE emerged [92]. First, while it is decreasing globally, the rate of SE’d workers is increasing in developed countries [92]. Second, SE appeared with new space (e.g., digital platforms), names (e.g., disguised wage work, ‘gig’ work, and contracting), sectors, and industries (e.g., creative industries). Third, there is an emerging ambiguity observed in the legal definition of SE as much as this term is increasingly popular [92]. This ambiguity or complexity of classification/misclassification is reinforced by newly emerging labour market traits and sectors, such as ICT based labour market, globalized labour market, and neoliberal labour market. For example, traditionally, ‘own account’ workers, such as agriculture, forestry, fishing, retail trade, and crafts are common SE’d workers over the world. Similarly, SE’d workers from the sectors, such as building and construction, road and transport, media (e.g., journalist and photographer), actors, musicians and performers in the entertainment industry are also common sectors of SE. However, the non-traditional sectors for SE’d workers, such as graphic design, music composition, and information technology (IT) specialist, and software developer are recent developments due to the advent of globalization and technological advancement. These ever-changing work arrangements make it difficult to identify who is SE’d. On the one hand, the Australian Bureau of Statistics tried to draw a line between independent contractors and other business operators in order to paint a simple picture for SE: they can either be employing or non-employing. According to the Australian Bureau of Statistics (ABS), the ‘independent contractors’ are owner operators who personally provide a service for clients under a commercial contract (e.g., a courier owner-driver contracted to perform a specific delivery run). The ‘other business operators’ are different from ‘independent contractors’ in terms of two factors: they provide the service directly to the public rather than under a client contract (e.g., a taxi operator); and/or they manage others to perform the service rather than provide the service personally (e.g., an owner-operator of a trucking fleet that spends more of their time managing other drivers than driving trucks). Despite these demarcations of definition, the ABS still argues that these categories remain unclear. For instance, if the courier owner-driver, mentioned above, worked in an ad hoc manner with different daily changing clients, they can be identified as both an independent contractor and an other business operator. In Australia, ultimately the status of worker–whether he/she is employee, self-employed, or independent contractor–has evolved into disputed and contestable cases before the courts. Small business or solo traders are also often understood as SE’d. In practice, the smallest businesses are likely to be operated and/or managed by someone who is SE’d. However, it is unclear what percentage of small business owners regard themselves as SE’d, and there is no agreed standard to define their size and traits to be SE. According to Australian Business Statistics, small businesses include firms that are non-employing, microbusinesses employing less than five people, and other small businesses employing less than 20 people. On the other hand, Statistics Canada (2015) has more clear-cut distinctions in this context: owners of incorporated and unincorporated business, farm, and professional practices are deemed as SE’d. The latter groups are also SE’d, though they do not own a business, such as babysitters. Incorporated groups may be of two types: those who have paid helping hands and those do not have such helpers. Statistics Canada (2015) also includes in SE’d groups those who help other family members’ business, farm or professional practices, without receiving salary/wages.

The self-employed include working owners of an incorporated business, farm or professional practice, or working owners of an unincorporated business, farm or professional practice. The latter group also includes self-employed workers who do not own a business (such as babysitters and newspaper carriers). Self-employed workers are further subdivided by those with or without paid help. Also included among the self-employed are unpaid family workers. They are persons who work without pay on a farm or in a business or professional practice owned and operated by another family member living in the same dwelling. They represented in 2011 about 1% of the self-employed. To put the analysis succinctly, Australia seems conservative in demarcating the multidimensional features of SE. Although it distinguished independent contractor from the business operator, it still remains unclear. However, Statistics Canada is liberal to fragments the SE, by clearly defining incorporated and unincorporated SE.

Finally, we view, across all the national contexts and differences, the SE through a broader lens, as individuals who work for themselves instead of working for others like paid workers. Many may work alone, but others may have their own small business with or without employees. In this sense, there is an inevitable overlap between employers, self-employees, and employees. In short, SE is a diverse work arrangement, encompassing occupations ranging from highly paid professionals or billionaire entrepreneurs to low-skilled workers operating a business on their own.

### 4.2. Relationship between Misclassification of SE and Social Security Systems

SE’d workers are often misclassified because employers seek to reduce legal commitments and compensation. The potential (mis)classification of workers in dependent employment relationships such as SE’d has been described by socio-legal scholars, as well as the European Commission and the International Labour Organization, due to the rising ‘gig’ economy in certain industries, such as construction industries [44,93]. Not surprisingly, rights and obligations are less entertaining for SE’d workers than for regular employees [10,44,93,94]. In addition, sham contractors is a term which is widely used to misclassify SE’d workers. This refers to people who are wrongly regarded as independent contractors and who are identical to employees [93]. This problem is recognised by some authorities. For instance, the Australian NSW Road Transport Authority has prescribed a substantive system of collective rights in order to resolve disputes overcompensations, introducing a new Road Safety Remuneration Tribunal, where minimum standards can be set for all truck drivers, whether they are employees or SE’d [95]. In a nutshell, if employers misclassify employees as self-employed/independent contractors, in turn, they are denied access to critical benefits and protections in Ontario, Canada, and workers agree because they want to ensure certain income [2]. The Australian (NSW) labour market encounters similar experiences.

### 4.3. Existing Social Security Systems for Workers and SE’d Workers: Ontario and NSW

Both Ontario and NSW have multiple mediums to support their citizens as well as workers in terms of government and non-government agencies by involving different stakeholders, such as hospital, ministries of governments, insurance boards and companies (Table 5). Generally, Australian and Canadian social security systems are different from each other because, unlike Canada, Australian systems do not depend on social insurance or the workers’ previous contributions, and their system relies on general government revenue [76].

#### 4.3.1. Supports Available to People Regardless of Employment Status

In Ontario, Canada, people, regardless of prior employment status, are entitled to get support from the Ontario Disability Support Program (ODSP), if they are 18 years and older, disabled and need support to meet living expenses, and their family income and assets are below a cut-off line. As such, eligibility is assessed both financially and medically. ODSP offers financial assistance to claimants and their family for essential living expenses, prescription drugs, vision care, help to find jobs and training to continue their jobs. Similarly, ‘Ontario Works’ provides financial and employment assistance to people, regardless of the nature of the jobs, who are 16 years and older, and in need of meeting basic living expenses for themselves or their family (Ontario Ministry of Children, Community and Social Services, 2019). They are provided with financial assistance, including income support to help with the costs of basic needs, health benefits for clients and their families, and employment assistance to help clients find, prepare for, and keep a job.

In terms of Medicare, citizens and permanent residents in Ontario are entitled to the Ontario Health Insurance Plan (OHIP) in order to use medical facilities, cover appointments with doctors, hospital emergency rooms, medical tests and surgeries. Every Canadian citizen and permanent resident including their families, except people from Québec, which has its own plan, are entitled to have a Canada Pension Plan (CPP), covering partial replacement of earnings during retirement, disability or death. Benefits include a retirement pension, disability benefits, survivor’s pension, death benefits, and children’s benefits. To sum up, ODSP does not require contributions from workers, but it is means-tested, whereas OHIP and CPP are not means-tested. Workers have to contribute to a fund to be eligible for CPP, but not for OHIP.

Regardless of place of injury either in the course of work or outside of work, in NSW, Australia, anyone can have access to supports from icare, self-insurance, and specialised insurance, which are managed/implemented by SIRA (State Insurance Regulatory Authority). These supports are provided to all Australian residents across social assistance and mandatory occupational pension systems, such as old age pension, disability pension, survivor’s pension. The social assistance (cash sickness benefits) and universal (medical benefits) systems cover sickness and maternity benefits, temporary disability benefits, permanent disability benefits, and workers’ medical benefits, and unemployment and family allowances, involving compulsory insurance with a public or private carrier under different schemes established and run by state and territory governments.

However, people in NSW, including the SE’d, are required to pay a Compulsory Third Party Premium (CTP) when a vehicle is registered for motor accident insurance, which is managed by SIRA under a Compulsory Third Party (CTP) scheme, which covers injury involving motor vehicles. The benefits coverage of this scheme includes compensation for people who are killed or injured. Compensation can also include hospital, medical and rehabilitation costs, loss of earnings, and pain and suffering. Some aspects of compensation are reliant on establishing fault by another party and some are payable regardless of fault. The third-party insurance component of the scheme (CTP) is underwritten by five insurers. Insurer pricing and behaviour is monitored and regulated by SIRA. Finally, the National Disability Insurance Scheme (NDIS) is also a federal government funded program for disabled (irrespective of causes) people from 7 to 64 years old living in Australia with permanent and significant disability, and it may be the main supplier of benefits, or additional to other state funded supports. Overall, most government benefits are income-tested and asset-tested, implying that workers’ entitlements reduce as resources increase [76].

#### 4.3.2. Supports Available That Self-Employed Can Opt into

In Ontario, an Employment Insurance special benefits (EI) exist, which SE’d workers in Ontario can opt into if they choose to register with CEIS (Canada Employment Insurance Commission). This provides benefits, one year after registering and paying monthly premiums, including maternity, parental, sickness, compassionate care, family caregiver for children, and family caregiver for adults (Government of Canada, 2013). In this case, a SE’d worker who claims for compensation may receive up to 55% of his/her average weekly pay up to a maximum annual limit. However, if business revenue is generated during their leave, the funds are reduced accordingly (Service Canada, 2014). According to a report by the Canada Employment Insurance Commission (2014), SE’d women between the ages of 25 and 44 years old made 90.4% of all special benefits claims, mostly for maternity and parental benefits. According to Hilbrecht [30], there are some evidence that a significant number of entitled SE’d workers do not seek and claim compensations mainly due to lack of information about the supports [30].

In Australia, including NSW, SE’d workers can opt into the work injury scheme if they voluntarily participate by paying premiums for self-insurance. This covers temporary and permanent disability benefits, and workers’ medical benefits as well as unemployment and family allowances. In addition, SE’d workers in NSW can buy personal injury/accident insurance, though it is not connected to CTP. In addition to other injury, it may cover insurer for injury in the event of a motor vehicle accident, regardless the fault. It may also cover gaps or limitations in the private health insurance shows.

Generally, it is still challenging to define how many SE’d workers are under coverage of government and private supports because the existing evidence pertinent to SE and compensation regimes is scarce, conflicting, and partial [17]. There is evidence that precarious workers, including SE’d, are less likely to make compensations claims, compared to regular employees [4]. SE is one of the four categories of employment-unskilled workers, occupationally mobile, SE’d, and geographically isolated-in terms of the highest underreporting for compensation claims, while 27% injured workers did not submit claims for compensation, as found in a study in Queensland, Australia, for example [4]. The Australian Bureau of Statistics also investigated why a large number of injured workers do not claim for compensations, and fund that 14.4% of workers are SE’d and they think they are not eligible for compensations [96]. In some Australian jurisdictions, there are very uneven systems of coverage for SE. For example, some SE’d workers are included in compulsory coverage, but other forms of SE have the option of voluntary cover, private accident insurance, or nothing. Of importance, around 20% of SE’d workers have no coverage, whatever their pattern of work [4]. The situation is more complicated in Queensland where compulsory coverage for some SE’d workers and a voluntary option for others was curtailed in 1997 [4]. In addition, occupational health and safety statistics mask the statistics of SE’d workers in mining industry in Australia [4]. Thus, SE’d workers are excluded from workers compensation claims, as well as those who have coverage but do not lodge claims because of ignorance, lack of information, financial pressure to keep the job [24]. Some studies also found that under-insurance and non-payment are responsible for being reported in the documents (e.g., NSW, Australia), and it is done intentionally in order to manipulate the classification of work and evade the tax and compensation [4].

To sum up, SE’d workers in NSW have more access to schemes based on voluntary participation than do these workers in Ontario. As such, supports are provided in Ontario irrespective of workers’ employment status, whereas some are means-tested (e.g., ODSP) and some schemes requires contributions from the workers (e.g., CPP). Similarly, workers in NSW regardless of their employment status have also access to several types of social supports. Of these, some of the schemes expect contributions from the workers (e.g., motor accident insurance). However, Ontario has limited provisions including the EI special benefits program, which provides SE’d people with a significant number of benefits in return for paying a premium, though it fails to attract low-earning SE’d workers because they cannot afford it with the high rate of premiums. In terms of mandatory schemes, both jurisdictions have multiple alternatives, but each provide limited provisions for SE’d due largely to complicated eligibility criteria.

## 5. Discussion

Currently, key challenges with SE are in its definition, conceptualization, and classification. Mounting evidence shows that SE is often misclassified and mistakenly defined [2,4,49,54,62,91,92,97]. Consistent specification of the status of SE across employment frameworks and classifications is needed in order to design eligibility requirements for social supports and compensation for a work injury or disability. At the same time, the heterogeneity of SE’s needs to be recognised [53]. For instance, a growing problem exists with organisations, such as digital employment platforms, classifying their workers as SE’d for purposes of tax and insurance premium evasion. Our study reaffirms the need to reconsider the ambiguous position of SE’d in the current labour market, as the SE’d include a range from low-income digital platform workers to successful entrepreneurs [98]. As most government bodies have homogenised support systems wherein SE’d are recognised as only one category of worker, deserving SE’d workers become deprived of government supports when they are in need. Our study found that the current ‘objective’ evidence framing who is SE’d overlooks the push/pull factors that are critical to understanding their positioning in the SE labour market. For instance, workers may be ‘pushed’ in by lack of employment alternatives; and they might be ‘pulled’ in by the lure of neoliberal notions of freedom and autonomy [3]. In this way, the labels of ‘autonomy’ and ‘healthier’ are not realistic for SE’d workers because the conventional measurement and assessment of well-being of SE’d workers overlooks the diversity of SE and self-exploitation [53,99]. Against this backdrop, a central question is ubiquitous: who seeks government supports? The answer to this question lies in a robust understanding of the diversity of SE’d workers, as paramount for better (re)form policies in order to provide appropriate social protection for SE’d [10,100]. However, a barrier to accomplishing this work is a dearth of data related to SE.

To date, it seems that policies in Canada and Australia continue to visualize SE’d workers as the highly paid variety who may not need financial support when ill or injured. However, many studies have documented that this assertion about SE’d workers is an over-generalization and refers to a group of people who are financially prosperous, younger and highly educated, and who became SE’d for opportunity rather than necessity [7,53,100]. In this context, we argue that a significant number of SE’d workers living in Canada, Australia, and elsewhere are poorly paid and need income support during their absence from work due to injury and sickness. The invisibility of these precarious SE’d workers in policy is amplified by their vague status in policy formulations [3,100,101]. In addition, our study illustrates a strong relationship between precarious jobs and poorer health outcomes [32,102], and numerous social costs [15,33]. For example, SE’d workers are at higher risk for certain diseases compared to salaried workers [3,7,17]. However, this ‘employment type and health’ interplay is not always straightforward; rather, it is subject to the type of welfare state. For example, a systematic review suggests that Scandinavian welfare regimes show better or equal health outcomes for precarious workers compared to their counterparts (salaried, permanent employees), whereas precarious workers from other welfare regimes (e.g., Bismarckian, Southern European, Anglo-Saxon, Eastern European, and East Asian) show worse health outcomes compared to salaried and permanent employees [37,69,103]. Although Canada and Australia are well-developed welfare states, several studies demonstrate that precarious employment, including SE, plays a pivotal adverse role on people’s health and well-being [31,104,105,106].

Our review reveals that both Ontario and NSW have limited social security provisions for SE’d workers when injured, ill or out of work (Table 6). In Ontario, SE’d workers are supported under the systems of the Ontario Disability Support Program, Ontario Works, Ontario Health Insurance Plan (OHIP), Workplace Safety and Insurance (for construction workers only), Canada Pension Plan, and Employment Insurance special benefits. However, there is uneven accessibility to available supports. For example, ODSP is means-tested, whereas OHIP and CPP are not. Because people in Ontario have to contribute to a fund to be eligible for CPP and EI special benefits, in practice this means that many low-earning SE’d workers, such as ‘gig’ workers, do not participate because they cannot afford the premiums [100]. As such, these ‘gig’ workers are neither able to pay the premium nor be eligible for government accommodations [69]. This is a potential threat to the Canadian welfare state. Similar challenges exist elsewhere. For example, in Spain, according to Corujo [107], ‘Uberisation’ of work devastated labour and social security regulation, making the state powerless to undermine the political, legal, and financial foundations of welfare states. One more gap identified in our review is that SE’d employed people are not always aware of existing government provided support [2,30]. Indeed, other Canadian studies found that when SE’d workers need extra support, they rely heavily on informal support systems, such family members and friends [30,100,108]. Although some SE’d rely on personal savings [30,98], many lower earning SE’d workers cannot save enough to support non-working time [30]. In NSW, most of the supports, such as old age benefits, disability benefits, unemployment allowance etc., include SE’d workers, together with compulsory premiums to access work injury and personal injury/accident insurance. Overall, the Australian social security systems for workers, including SE, is remarkably different from other OECD countries, including Canada because Australian systems do not depend on workers’ previous contributions to be eligible for supports [76]. In our view, these differences might create bureaucratic complications for Canadian claimants, irrespective of employment status.

Both jurisdictions, NSW and Ontario, have strengths and drawbacks in terms of support systems available for SE’d workers. On one hand, Ontario’s SE’d- focused special EI is comprehensive, and covers maternity, parental, sickness, compassionate care, family caregiver for children, and family caregiver for adults (Government of Canada, 2013). On the other hand, most of NSW’s systems are narrow and constrained by multiple conditions. For example, NSW’s workers’ compensation and work injury covers only injury, not sickness or disease, and the injury needs to be caused by work. It is noteworthy that proving benefits for work-related injury for SE’d people is challenging because their working relationships and arrangements often blur, unlike those of many regular employees. For example, a SE’d person with a home office may have difficulty distinguishing a home-related versus a work-related accident. Another important difference between the two jurisdictions is that SE’d workers in NSW are entitled to apply for unemployment allowance, which is solely provided by government, whereas this is not possible for SE’d workers in Ontario. Similarly, SE’d people in NSW who have limited income can apply for sickness and maternity benefits, and family allowances. However, SE’d workers in NSW are excluded from other supports, such as old age pension, disability pension, survivor’s pension.

SE’d workers can pay for private insurance with sickness and injury coverage in Ontario. However, when the WSIB imposed mandatory insurance on the SE’d construction workers in Ontario in 2013, they encountered protests from independent contractors who did not want to be required to pay this insurance premium that was more costly than what they had been paying for private insurance and that did not cover non-work-related illness and injury [109]. In our view, however, this overlooks the reality that increasing numbers of SE’d workers are low earning and need income and health protection [109]. Government provided schemes provide stronger protection than private ones, such as workers’ compensation providing income support through the course of life, if needed. Further, several Eurocentric reports expressed concern that private insurance may exacerbate poverty and inequality, including gender gaps, because it has a limited capacity for ‘risk pooling and redistribution’ compared to social insurance [7,98,100,110,111]. In this context, where support systems are lacking for SE’d workers, they can encounter very adverse situations. In addition, studies show that the precarious employment position of SE’d workers adversely affects their important life decisions, such as marriage and childbearing [98]. Overall, there are ample drawbacks of SE that may outweigh the benefits (e.g., economic growth, flexible schedule), that can affect the quality of family life (e.g., work-life balance, irregular or anti-social work hours, fewer vacation and sick days, negotiating workload), if they have limited access to statutory and social benefits [30]. These concerns, pertinent to social protections, and the future of SE, have also been raised in empirical research in Canada [3,7,100].

## 6. Conclusions and Recommendations

Regardless of the segment of SE, be it independent contractor, entrepreneurship, small business, startup, unlike employees, the issue of supporting SE’d workers during injury and sickness is an ignored discourse in Canada and Australia. There is a gulf between how the number of SE’d workers are ballooning against the backdrop of the ‘gig’ economy and how these rising working populations lack attention in social security systems in Ontario and NSW. Policies in both jurisdictions appear to be based on the traditional picture of prosperous, well-organized SE’d workers not needing support from the state. However, this is an overgeneralization and a hyper-reality because at present tens of thousands of low paying SE’d workers strive to lead a decent life. Undoubtedly, they face very difficult circumstances when they have to be away from work due to injury or sickness, as this strata of the SE’d population generally cannot afford private insurance. In fact, at present, compensation for SE’d workers in both Ontario and NSW remains deceptive. Work is needed at both the policy and practice level to incorporate the voices of SE’d workers into compensation. Our comparative discussion leads us toward conclusions about what might need to be done to continue with unmasking the illusion of the traditional well-to-do self-employed worker:

(i) Although ‘Employment Insurance special benefits’ in Canada are not always used by SE’d workers in Canada due to the financial burden of premium payments, it nonetheless provides an example of a coverage system for SE’d workers that provides temporary income supports for parental, sickness or compassionate support leave etc. This is one way in which SE’d workers are recognized as a cohort. Hence, in the sense of equity, SE’d workers in NSW, Australia, might be treated in a similar manner, but after revisiting the issue of premiums.
(ii) Basic income policies may be a solution to providing a basic social safety net to SE’d people, among others. An advantage of this approach is that it draws on the general tax fund rather than relying on taxing incomes of low-wage SE’d people, who are already income insecure [71,112].
(iii) All workers, whether SE’d or not, should be covered by workers’ compensation regimes. Digital platforms such as Uber should be required to pay into this scheme.
(iv) For both jurisdictions, emergency income supports can be introduced for SE’d workers so that they can be supported when facing emergency circumstances, including but not limited to natural disaster, pandemic, injury/sickness. In this context, for example, COVID emergency benefits in Canada (CERB, Canada) was a successful program to address and protect SE’d workers.
(v) Against the backdrop of a changing labour market in the digital age, SE is inevitable and obvious. A premise guiding policymaking is that SE’d workers should not be at a social security disadvantage relative to employees.
(vi) Governments should create explicit policy to deal with SE’d and precarious workers to remove grey zones and clarify eligibility for compensation.
(vii) As women and recent immigrants are more prone to be SE’d workers in recent years, childcare for the SE’d deserves special policy attention.
(viii) Underreporting of compensation claims is a big issue for the labour market and social safety net policies. A strong social mobilization program may be required in order to reduce underreporting.
(ix) A social supports literacy campaign may be introduced by both jurisdictions, using mass media or social media, because most of the SE’d workers in practice are not aware of the available supports systems to which they are entitled. However, there are still some support systems available for the SE’d workers in both jurisdictions.
(x) In the case of both jurisdictions, SE’d workers, irrespective of the sector of work, platforms (digital or offline), structure of working relations (solo or paid employees), size of the business/professional clients (small or solo traders) need to be given access to ‘collective bargaining’. These rights should be granted whenever necessary to prevent the contracting party with the dominant bargaining position from exercising a compression of labour standards [70]. In this context, both jurisdictions need to become ‘open’ to reforming the existing employment standards or other regulatory protocols pertinent to employment if necessary. As such, trade unions and businesses agree on a series of workers’ prerogatives, leading to the creation of a level playing field in terms of labour costs and ensuring clients that a company’s success does not depend on lowering working conditions [70].

We are aware of a number of limitations of our study. First, we were dogged by the dearth of data around SE’d workers for both countries. It was a challenge to sort the data for SE’d workers from other precarious workers because most of the documents overlap these segments of employment. In short, we agree with several researchers that SE is poorly documented and understood. Second, in both Ontario and NSW, the labour market is undergoing rapid change and development at both policy and practice levels. Therefore, what we have written about in both places is not static; nevertheless, we argue that the broad themes emerging from our work will be relevant in both places for a significant time to come. Apart from the established databases for scholarly articles and documents, we relied on Google’s search engine to capture grey literature and ongoing government data. As the outcome of Google searches are filtered, we worked diligently to sort out the relevant documents. Despite these limitations, consideration of the issues that emerged from our description and analysis of identifying SE’d workers and compensation or supports for absence for work due to sickness and injury policy and practice in both countries will, we hope, support policy makers, people working in administering workers and compensations, researchers in their task of moving toward a sustainable compensation policy, and the imperative of tackling the gaps in the existing systems.

## Figures and Tables

**Table 1:** Description of literature identified by the non-systematic search.

Author, Year (Reference)	Main Focus	Method	Country, Sector
L.C.O., Ontario [2]	Providing comprehensive provincial strategy and recommendations based on Identifying vulnerable and precarious workers, employment standards, and related legislative reformations	Review/policy analysis/classical legal analysis	Canada, any type
Wall [1]	Examining the experiences of SE’d nurses as self-employment in professional caring work.	Qualitative	Canada, Nurse
Bögenhold [49]	Elaborating the heterogeneity of SE	Review	Global, any type
Weil [50]	Providing an overview of core elements comprising fissuring workplaces.	Review	Global, any type
Yssad [14]	Providing statistical overview of SE	Review	Canada, any type
(ASFA) [13]	Providing demographic and economic characteristics of SE’d workers.	Review	Australia, any type
Facey and Eakin [9]	Developing a framework for conceptualizing contingent work and its relationship to health.	Review	Global, any type
OECD [10]	Discussing how labour market regulations can protect non-standard workers.	Review	OECD countries Any type
Apouey [11]	Examining the effect of both self and temporary employment on mental health in the UK.	Review	UK, any type
Taylor, Marsh, Nicol and Broadbent [5]	Providing a comprehensive overview/review of modern working practices.	Review	UK, any type
Nordenmark, et al. [51]	Showing linkage between job control and demands, the work-life balance, and wellbeing among SE’d men and women.	Quantitative	26 European countries, any type
Kautonen, Kibler and Minniti [23]	Examining how late-career transitions from org employment to entrepreneurship impact the returns from the monetary and quality of life.	Quantitative	UK, any type
Nordenmark, et al. [52]	Examining the occurrence of sickness presenteeism among the organizationally employed SE and any differences can be explained by higher work demands among the SE’d.	Quantitative	European Union, any type
Bujacz, et al. [53]	Examining and identifying the profiles of the SE’d taking into account different well-being indicators.	Quantitative	Europe, any type
Vermeylen, Wilkens, Biletta and Fromm [44]	Identifying heterogeneity of SE’d in terms of wide-ranging attitudes, income levels, and health and well-being among this diverse group.	Review	European Union, any type
Fudge [54]	Reviewing labour protection for SE’d workers	Review	Canada, any type
Dahl, Nielsen and Mojtabai [33]	Investigating how entering entrepreneurship affects the people involved.	Quantitative	Denmark, any type
Stephan and Roesler [55]	Comparing entrepreneurs’ health with employees’ health in a national representative sample.	Quantitative	German, any type
Bennaars [56]	Assessing the EU concept of a worker, self-employed, dependent self-employment, and false self-employment, EU legislation providing social protection for the SE’d.	Review	European Union, any type
Boeri, et al. [57]	Documenting features of solo SE, SE with employees, employment, and unemployment.	Review	OECD countries, any type
Dixon-Woods, et al. [58]	Focusing on a reflexive account of an attempt to conduct an interpretive review of the literature on access to healthcare by vulnerable groups in the UK.	Review	UK, any type
Hudon, et al. [59]	Comparing critical literature on the practices of first-line providers for workers with musculoskeletal injuries.	Review	Canada, United States, Australia, any type
Cassidy [60]	Understanding how to deal with the solitude of SE.	Newspaper article	UK, any type
MacEachen [61]	Examining occupational health and safety conditions of Uber work.	Qualitative	Canada, Uber drivers
Thörnquist [62]	Discussing the problem of false (bogus) SE and other precarious forms of employment in the ‘grey area’ between genuine SE and subordinate employment.	Review	Sweden, construction, & cleaning
Behling and Harvey [48]	Examining how the co-evolution of employment status law and a sector-specific fiscal regime maps tightly onto the emergence of mass SE, as evidenced by the comparative labour market and sectoral statistics.	Quantitative	UK, construction
Bartel, et al. [63]	Focuses on ride-share drivers’ health risks on the job	Qualitative	Canada, rideshare
Tran and Sokas [64]	Addressing the needs of workers in non-traditional employment relationships.	Review	USA, Physicians
Bajwa, et al. [65]	Presenting a commentary on the implications of a globalized online platform labour market on the health of ‘gig’ workers in Canada and globally.	Review	Canada, gig workers
Browne [66]	Review on reform to worker compensation systems of NSW.	Review	Australia, any type
Lippel [67]	Identifying the impacts of compensation system characteristics on doctors in Quebec and Ontario.	Qualitative, Legal analysis	Canada, any type
Purse [68]	Identifying the trajectory of workers’ compensation in Australia.	Review	Australia, any type
Spasova, et al. [69]	Synthesising both statutory and effective access to social protection for people in non-standard employment and self-employment in Europe.	Review	Europe, any type
Rainone and Countouris [70]	This policy report discusses a possible reconfiguration of the coexistence between collective bargaining and competition law.	Policy brief	Europe, any type
Pasma and Regehr [71]	Constructing a model for basic income that is fair, effective, and feasible in Canada.	Policy analysis	Canada, any type
Busby and Muthukumaran [72]	Looking at the common meanings of precarious work in academic and policy research, by examining the trends in non-standard work in Canada.	Policy analysis	Canada, any type
Laflamme [73]	Examining how the new working relationships and related protection systems are addressed in the province of Canada) and the Australian OHS regimes.	Policy analysis	Canada, Australia, Any type
May [74]	Developing a definition of precarious employment and its indicators and identifying the role that precarious employment plays in the economy.	Policy analysis	Canada, any type
Lippel and Lötters [75]	A comparison of cause-based and disability-based income support systems	Review	Global, any type
Whiteford and Heron [76]	Assessing social protection systems for workers.	Review	Australia, any type

**Table 3:** Legal Frameworks addressing Self-employment.

Ontario, Canada	
**Labour Relations Act. 1995** The definition of employee under the Labour Relations Act includes dependent contractor: “*dependent contractor*” means a person, whether or not employed under a contract of employment, and whether or not furnishing tools, vehicles, equipment, machinery, material, or any other thing owned by the dependent contractor, who performs work or services for another person for compensation or reward on such terms and conditions that the dependent contractor is in a position of economic dependence upon, and under an obligation to perform duties for, that person more closely resembling the relationship of an employee than that of an independent contractor”	**WSIB, Ontario** *Independent operators (in construction):* WSIB consider a person an independent operator in construction sector if he/she is sole proprietor or sole executive officer of a corporation, and subject to performing Class G construction work, no employees, working as contractor or subcontractor for more than one person during an 18-month period, reporting as ‘self-employed’ to a government agency, like the Canada Revenue Agency. **Workplace Safety and Insurance Act, 1997** It defines “Worker” and “Employer”. “*Worker* means a person who has entered into or is employed under a contract of service or apprenticeship”.
**Employment Standards Act (ESA), 2000** It defines “Employee” and “Employer”. *“Employee*” includes, (a) a person, including an officer of a corporation, who performs work for an employer for wages, (b) a person who supplies services to an employer for wages, (c) a person who receives training from a person who is an employer, if the skill in which the person is being trained is a skill used by the employer’s employees, or (d) a person who is a homeworker, and includes a person who was an employee”. *No information provided about dependent contractor or self-employment.*	
**NSW, Australia**	
**Workplace Injury Management and Workers Compensation Act. 1998** No definition of SE **Workers Compensation Regulation. 2003** Define two categories of employers. But no definition of SE. **Workers Compensation Act. 1987** No definition of SE. **The Fair Work Act. 2009** The National Employment Standards (NES) cover 11 types of employees under National workplace relations system, but these talk nothing of SE. **The Industrial Relations Act. 1996, NSW** It broadens the definitions of employees, where SE’d can be accommodated: (1) in general definition, employee includes: (a) a person employed in any industry, whether on salary or wages or piece-work rates, or (b) any person taken to be an employee by subsection. (2) A person is not prevented from being an employee only because—(a) the person is working under a contract for labour only, or substantially for labour only, or (b) the person works part-time or on a casual basis, or (c) the person is the lessee of any tools or other implements of production, or(d) the person is an outworker, or (e) the person is paid wholly or partly by commission (such as a person working in the capacity of salesperson, commercial traveler or insurance agent). (3) Deemed employees: the persons described in Schedule 1 are taken to be employees for the purposes of this Act. Any person described in that Schedule as the employer of such an employee is taken to be the employer. (4) Exclusion: a person employed or engaged by his or her spouse, de facto partner or parent is not an employee for the purposes of this Act.	

**Table 4:** Different terms for self-employment.

Independent operator ‘Gig’ worker ‘Gig’ employment Entrepreneur Self-employment without employee Self-employment with employee Independent contractor Dependent contractor Disguised worker Bogus worker False Worker	Sham worker Own account self-employment Solo self-employment Stable own account self-employment Own boss employment Own boss worker Unincorporated self-employment Incorporated self-employment Dependent self-employment Economically dependent self-employment

**Table 5:** Government and Non-Government Supports for SE’d Workers following illness or injury.

Ontario, Canada	NSW, Australia
**Supports That Cover/Required for all SE’d Workers**	
Ontario Disability Support Program Ontario Works Ontario Health Insurance Plan (OHIP) Workplace Safety and Insurance (for construction workers only) Canada Pension Plan (Federal)	Old Age Pension Disability support pension (DSP) Survivor’s pension Sickness and maternity benefits Unemployment Family allowances Motor Accident Insurance (Compulsory Third Party) National Disability Insurance Scheme (NDSI)
**Supports That Are Available to SE’d Workers only if They opt in and pay a premium**	
Employment Insurance Special Benefits (federal) WSIB (for all occupations except construction)	Work injury Personal injury/accident insurance

**Table 6:** Key Supportive Policies.

Ontario, Canada	NSW, Australia
Ontario Disability Support Program Ontario Works Ontario Health Insurance Plan (OHIP) Workplace Safety and Insurance (for construction workers only) Canada Pension Plan (Federal) Employment Insurance Special Benefits (federal) WSIB (for all occupations except construction)	Old Age Pension Disability Support Pension (DSP) Survivor’s Pension Sickness and Maternity Benefits Unemployment Benefits Family Allowances Motor Accident Insurance National Disability Insurance Scheme (NDSI) Work Injury Personal Injury/Accident Insurance

## Data Availability

Not applicable.

## Appendix A

(*italic terms were used for the second search*): *Self employ*, *independent operator*, ‘*gig*’ *Work*, ‘*gig*’ *employ*, entrepreneur, employment without employ, *independent contract*, *dependent contract*, disguised work, bogus work, false work, *own account self-employ*, *solo self employ*, solo self-employ, *stable own account self-employ*, *own boss employ*, *own boss work*, *unincorporated self-employ*, *dependent self-employ*, *economically dependent self-employ*, health, injury, disability, impairment, stress, well-being, wellness, long and irregular working, flexible working schedule, work-life Balance, access to care, access to health care, body mass index, physical health, mental health, diabetes, high blood pressure, high cholesterol, arthritis, *return to work*, *RTW*, *work reintegration*, sick leave, pension, insurance, vocational rehabilitation, disability insurance, sickness absence, retirement disability pension, and public health insurance. *work disability policy*, *workers rehabilitate*, *occupational safety regulations*, *social safety net programs*, *Australia*, *Ontario*, *Canada*.
